# Psychosocial Adjustment and Sociometric Status in Primary Education: Gender Differences

**DOI:** 10.3389/fpsyg.2020.607274

**Published:** 2020-12-08

**Authors:** Alicia Muñoz-Silva, Cecilia De la Corte de la Corte, Bárbara Lorence-Lara, Manuel Sanchez-Garcia

**Affiliations:** ^1^Department of Social, Developmental and Educational Psychology, University of Huelva, Huelva, Spain; ^2^Department of Developmental and Educational Psychology, University of Sevilla, Sevilla, Spain; ^3^Department of Clinical and Experimental Psychology, University of Huelva, Huelva, Spain

**Keywords:** sociometric status, adjustment, behavioral problems, gender differences, education

## Abstract

The acceptance or rejection of classmates is one of the most widely recognized determinants of wellbeing in childhood. This study analyses psychosocial adjustment and sociometric status in primary education pupils, and possible differences by gender. A cross-sectional survey was undertaken in Huelva (Spain). The surveyed schools were selected using a stratified random sampling technique with both public and private elementary schools. Sample was composed of 247 4th grade students. Data revealed gender differences in psychosocial adjustment, particularly in terms of prosocial behavior in girls and behavioral problems in boys. Popular and rejected statuses presented opposing adjustment profiles, particularly in hyperactive symptoms and behavioral problems. When the sample was separated by gender, the differences between the types of status in emotional symptomatology and prosocial behavior disappeared. In addition, the differences between statuses were greater in boys, and were defined mainly by hyperactivity, whilst for girls these differences were more apparent in behavioral problems.

## Introduction

Worldwide, 10–20% of children and adolescents suffer from psychiatric disorders, but only one fifth are correctly diagnosed. Moreover, significant numbers of other children and young people have psychosocial adjustment problems that, whilst not meeting the diagnostic criteria of a mental disorder, are still a source of suffering and discomfort, both for themselves and for the people around them. These minors must also receive evaluation and help (Asociación Española de Neuropsiquiatría, 2009; Mundy et al., 2017). Furthermore, the early presence of emotional and/or behavioral problems increases the risk of developing a mental disorder in the later stages of development (Costello et al., 2003; Kim-Cohen et al., 2003; Widiger et al., 2009). Concretely, in late childhood — our work is focused on this developmental stage — behavioral and/or emotional disorders are particularly pronounced (Navarro-Pardo et al., 2012).

On the other hand, the differences between boys and girls in their psychological adjustment have been well studied. In general, boys show a greater number of externalizing disorders than girls (Wenar and Kerig, 2000; Navarro-Pardo et al., 2012). Moreover, and without ignoring the high presence of behavioral problems in girls during childhood and adolescence that has been found in some studies (Aláez et al., 2000), girls surpass boys in internalizing pathologies such as depressive and anxiety disorders (Reitz et al., 2005; Navarro-Pardo et al., 2012). It is noteworthy that these gender differences can be seen in different countries and cultures such as Australia, Belgium, China, Germany, Greece, Israel, Jamaica, Holland, Puerto Rico, Sweden, Thailand, and the United States (Crijnen et al., 1999).

From middle childhood to preadolescence the school context has been shown decisive for the psychosocial adjustment of children. And in this context, there are two determining factors: (a) the teacher-student relationships and (b) the peer relationships. The quality of teacher-student relationships has been shown to be a protective factor against increasing internalizing and/or externalizing problems while stimulating prosocial behavior in children (Longobardi et al., 2019, 2020). In this sense, some works point out that teachers play an important mediation role in conflicts between classmates by correcting some behaviors and encouraging others (Gastaldi et al., 2015). Similarly, a good quality teacher-student relationship is a protective factor against the risk of victimization or exclusion from the group (Marengo et al., 2018). The second factor – peer relationships in school context and its association with psychosocial adjustment – is a central topic of this work.

Relationships with peers at these ages play an important role in understanding the emotional and behavioral difficulties of these children. During these ages, children function in the presence of their peers for a large proportion of their day, making the peer environment very important for their development (Sroufe et al., 2005; Rubin et al., 2015). In particular, the acceptance or rejection of peers is one of the most widely recognized determinants of socio-personal development and adjustment (Buhs and Ladd, 2001; Bierman, 2004; Prinstein and La Greca, 2004; Brendgen et al., 2005; Rubin et al., 2006; Laursen et al., 2007; Mrug et al., 2012; Platt et al., 2013; Badenes-Ribera et al., 2019). Thus, the acceptance of peers is associated with greater sociability, self-efficacy, and adequate self-esteem, while the absence of such relationships or continued rejection are related to isolation, depression, low sociability, poor prosociality, and disruptive behavior. In particular, the classroom group is usually the scenario par excellence in which social relationships are studied at these ages (Newcomb et al., 1993) and there are various studies showing that the degree of acceptance and rejection of peers in the school environment is also associated with the development of academic life and scholarly competence (Buhs and Ladd, 2001; Gifford-Smith and Brownell, 2003; Wentzel, 2003).

Given the important repercussions of the acceptance or rejection of peers, it is not surprising that numerous investigations have focused on studying the social status that boys and girls occupy in classroom settings. Primarily because of children during elementary school years are embedded in an age stratified group for such a large portion of their time (Hawley and Bower, 2018). One of the most widely employed ways of determining the acceptance or rejection of peers is through the analysis of sociometric status, studied through various methodologies, of which the sociometric technique developed in the 1930s by Moreno is particularly prominent and is the most widely chosen procedure (Jiang and Cillessen, 2005). The application of this technique allows us to obtain the following profiles: average, popular, rejected, neglected and controversial. Average status group is composed by children receiving a moderate number of likes and dislikes and it is the reference group with whom the more extreme groups are compared. Popular children have high liking scores and low disliking scores. Rejected are actively disliked by their peers while neglected are simply barely nominated by their peers as liked or disliked. Finally, controversial children receive both high liking and disliking scores (Coie et al., 1982).

In Spain, the most common sociometric status in school children is average, followed by neglected, rejected, popular, and controversial (García et al., 2013). The stability of this status over the years was examined in the study of Jiang and Cillessen (2005), with the older boys and girls showing more stability than the younger children. The stability of sociometric status over time is special relevant because strengthen the potential of social status on children outcomes (Geukes et al., 2018; Ilmarinen et al., 2019).

Similarly, the association between sociometric status and personal adjustment is well documented (Gest et al., 2001; Bierman, 2004; Geukes et al., 2018; Ilmarinen et al., 2019). In this regard, it is worth noting that there are a greater number of studies that examine sociometric status in relation to externalizing problems as opposed to internalizing problems. In general, rejected children, in contrast with their more popular peers, present more unfavorable indicators of internal and external adjustment (Newcomb et al., 1993; LaFontana and Cillessen, 2002) whilst even less prosociality has been observed in children with this status compared with those of other status categories (Wentzel, 2003; Plazas et al., 2010). Antisocial minors (Plazas et al., 2010) and those with hyperactive symptoms (Mrug et al., 2012; Ros and Graziano, 2018) are more rejected by their peers. For this reason, children with hyperactivity tend to be more highly represented in the rejected category. The findings, however, are not so clear when studying aggressiveness, where it has been found that the status of aggressors depends on the type of aggression that is predominantly used when interacting with their peers (Karmen and Tefan, 2013). Regarding internalizing problems, it has been shown that anxious and/or depressive symptomatology appears to maintain and exacerbate social rejection (Newcomb et al., 1993; Brendgen et al., 2005). Taken together, it appears that two conflicting statuses can be observed according to the adjustment characteristics of the children: popular and rejected. Further, average, and even neglected children have received less attention because these are sociometric profiles with adjustment problems that are of relatively little concern. Finally, in spite of the fact that there are both similarities and differences between the characteristics of the rejected and controversial statuses, the latter status is one of the least studied due to its low representativeness in groups and instability over time (Newcomb et al., 1993).

The high prevalence of psychosocial adjustment problems in childhood, together with its link with sociometric typologies explains the interest in these types of studies. During primary education, children are increasingly concerned about their own social status within their closest peer group, i.e., their group-class. In addition, on the basis of developmental psychology studies it is known that there are differences in the social adjustment of boys and girls at these ages, and these must be taken into account (LaFontana and Cillessen, 2002). It is also known that until the adolescence, gender segregation dominates the socialization of boys and girls (Percer, 2002). Other studies have found differences between boys and girls in terms of the degree of acceptance or rejection they receive from their peers, with boys being most represented in the rejected status category (Plazas et al., 2010). However, relatively few studies have examined whether the association between sociometric status and emotional and behavioral symptomatology varies according to gender (Brendgen et al., 2005; Escobar et al., 2010).

Thus, the present research specifically aims to analyze the psychosocial adjustment of children, their sociometric status, and the possible relationships between these variables in a sample of primary school students, whilst exploring how this association may vary according to gender. The ultimate goal of this work is to understand the factors which predict the positive adaptation of children and adolescents to their school environments. This will, in turn, inform the development of intervention programs focused on the most important factors for promoting the psychological wellbeing of pupils, both emotionally and personally as well as in terms of social and peer relations.

## Materials and Methods

### Participants

The study population was 4th grade primary school students (around 9 years of age) in Huelva (Spain). The tests were administered in six educational centers, with a final sample of 247 students composed of 112 boys (45.3%) and 135 girls (54.7%). The sample, therefore, represents 14.26% of the population enrolled in the 4th year of primary school in Huelva. We can assume that the sample is representative of the population (1,732 students) with a confidence level of 95%, and maximum estimation error of 5.7%, assuming an average effect size and a power of 99%. The age of the participants ranged between 8 and 10 years, with an average of 9 years (*SD* = 0.343).

The sample was selected using a stratification procedure grouping the schools according to their ownership (public or private). Within each of the strata we decided to apply the tests in as many centers as possible, using the following criteria:

- Pupils enrolled in the fourth grade of primary school (both girls and boys).
- Acceptance of the research by the educational center.
- Written informed consent of the parents/guardians of the children.

Table 1 shows the socio-demographic characteristics of the sample. Only children who were unable to attend any of the 2 days used for fieldwork in each school center were excluded from the sample.

**TABLE 1:** Socio-demographic characteristics of the sample (*N* = 247).

	**Distribution**
**Gender**	
Boys	112 (45.3%)
Girls	135 (54.7%)
**Ownership of the center**	
Public	120 (48.6%)
Private	127 (51.4%)
**Age**	Mean (S.D.) = 9 (0.343)
8 years	15 (6.1%)
9 years	218 (88.3%)
10 years	14 (5.7%)

### Instruments

Several informants participated in this study. The students of this study reported of their sex, gender (socio-demographic data), and sociometric status; and the teachers informed about these pupils’ psychosocial adjustment.

#### Socio-Demographic Data

Age and gender of the pupils.

#### Sociometric Status

This was obtained using the Peer-Nomination Questionnaire (García-Bacete, 2008). The questions asked were: “Who are the THREE members of your class that you choose as BEST FRIENDS?,” and “Who are the THREE members of your class that you like LEAST AS FRIENDS?” From these two items four standardized scores were calculated for each participant: Total nominations as “Best friend,” Total nominations as “Worst friend,” Social impact, and Social preference. From these four standardized scores, participants were categorized into the five sociometric status groups already described (Coie et al., 1982): Popular, Rejected, Neglected, Controversial, and Average.

#### Psychosocial Adjustment

This was evaluated using the Strengths and Difficulties Questionnaire (SDQ-Cas; Goodman, 2001) a validated brief mental health-screening questionnaire, designed to evaluate the behavior of children and adolescents between 3 and 16 years of age from the perspective of parents and teachers. In this work we used the version for teachers in Spanish (taken from^1^), which is composed of 25 items distributed into five subscales with five items each: Emotional Problems (α = 0.66, in this sample), Conduct Problems (α = 0.73), Hyperactivity (α = 0.88), Peer problems (α = 0.69) and Prosocial behavior (α = 0.84). Scores on each one of the items vary between 0 “Not true” and 2 “Absolutely true.” For each of the five scales the score can vary from 0 to 10. We also used the grouping into two second-order factors that was implemented by the authors of the scale (Goodman et al., 2010), obtaining a distinct score for internalizing symptomatology (α = 0.88), which is composed of Emotional Problems and Peer Problems, and externalizing symptomatology (α = 0.77), composed of Hyperactivity and Conduct Problems.

### Procedure

All procedures performed in this study were in accordance with the ethical standards of the institutional and/or national research committee and with the 1964 Helsinki declaration (protocol of Helsinky) and its later amendments or comparable ethical standards. Written informed consent was obtained from parents of all minors participants included in the study after thoroughly reading and fully understanding the information. A copy of the signature page was retained. No patients were included in this study and no ethics committee approval was needed.

The first step in the present investigation was to conduct an interview with the management team of each school to explain the methodology of the study. They were given specific information about the objectives of the research, making clear that the aim was to conduct a more in-depth search for variables that might improve the social adaptation of children, thus favoring the environment and welfare of the students in the educational centers. They were given a letter formally requesting collaboration with the study. After obtaining the consent of the center, a letter was written to the parents of the students where they were informed that a research team from the University of Huelva had requested the assistance of the school to conduct a study that aimed to evaluate the adaptation of children in their classrooms. In this letter, parents were informed that the tests were to be applied in the classroom and during school hours, and the duration was specified. In the same letter, the signed authorization of the parents/guardians was requested so that their child could be included in the study. Finally, each school appointed a 4th grade primary tutor to be present in each classroom when the tests were administered, accompanying the person who evaluated the students to ensure that pre-established class routines, including break times, were respected.

### Data Analysis

All the statistical analyzes were carried out using SPSS v. 20.0. Firstly, we examined the presence of missing data, checked erroneous data, and all that is implied by the purification of data through this statistical program. Secondly, we computed descriptive statistics (i.e., mean and standard deviation) for the study variables. The internal consistency of the subscale scores of the SDQ was also verified by Cronbach’s alpha coefficient. We analyzed the relationships between psychosocial adjustment and gender (*t*-test), and between psychosocial adjustment and sociometric status (one-way ANOVAs). For these analyzes, we estimate the effect size (Cohen, 1988, 1992): Cohen’s *d* values from | 0.20| to | 0.49| represent small effect sizes; those ranging from | 0.50| to | 0.79| reflect medium effect sizes; and values of | 0.80| or greater indicate large effect sizes. Eta-squared values from | 0.01| to | 0.05| represent small effect sizes; those ranging from | 0.06| to | 0.13| reflect medium effect sizes; and values of | 0.14| or greater indicate large effect sizes. Finally, we used chi-square test to analyze the relationships between sociometric status and gender.

## Results

### Psychosocial Adjustment and Sociometric Status of Primary Education Pupils

Table 2 shows the descriptive data for each of the first and second order subscales that make up the SDQ instrument. In general, the participants show more externalizing than internalizing problems [*t*_(__1_,_246__)_ = 7; *p* < 0.001; Cohen’s *d* = 0.45]. The scores obtained on the first-order subscales (emotional problems, peer problems, conduct problems, hyperactivity, and prosociality) present averages that fall within normality, as established in the correction of the scale (Goodman, 2001).

**TABLE 2:** Descriptive Statistics for the SDQ Subscales by Gender.

	**Total**	**Boys**	**Girls**	***t***	***d***
			
	***M (SD)***	***M (SD)***	***M (SD)***		
Total problems	7.53 (6.83)	8.46 (7.13)	6.75 (6.50)	1.97*	0.25
Internalizing problems	2.91 (3.12)	3.15 (3.04)	2.72 (3.17)	1.08	0.14
Emotional problems	1.59 (1.85)	1.76 (1.89)	1.44 (1.80)	1.33	0.17
Peer problems	1.33 (1.75)	1.39 (1.72)	1.27 (1.78)	0.53	0.07
Externalizing problems	4.61 (4.57)	5.31 (5.05)	4.03 (4.05)	2.21*	0.28
Conduct problems	1.36 (1.85)	1.67 (2.11)	1.10 (1.55)	2.44*	0.31
Hyperactivity	3.26 (3.11)	3.64 (3.31)	2.93 (2.90)	1.79	0.23
Prosociality	8.52 (1.93)	8.20 (2.16)	8.79 (1.68)	−2.40*	0.31

*p < 0.05; t = Student’s t-test; d = absolute value of Cohen’s d effect size.

The comparisons of means by gender reveal statistically significant differences in terms of Total Problems, these being more frequent in boys compared with girls (see Table 2). Specifically, these are the externalizing problems, with conduct problems being particularly highlighted in the boys. In contrast, girls score higher than boys on the prosocial scale.

The technique of peer nomination allowed for identifying the social status of each child in his/her classroom group (see Table 3). The percentage of pupils with average status exceeds 50% of the total sample, whilst the statuses of neglected, rejected, and popular all stand out to a lesser extent, with the controversial status being the least represented category.

**TABLE 3:** Comparison of the percentages of pupils in each status category according to gender.

	**Total *N* (%)**	**Boys % (Z)**	**Girls % (Z)**
Popular	32 (13.0%)	14.3 (0.6)	11.9 (−0.6)
Rejected	36 (14.6%)	17.9 (1.3)	11.9 (−1.3)
Neglected	42 (17.0%)	22.3 (2.0)	12.6 (−2.0)
Controversial	10 (4.0%)	2.7 (−1.0)	5.2 (1.0)
Average	127 (51.4%)	42.9 (−2.5)	58.5 (2.5)

*Pearson Chi-square = 9.02, degrees of freedom = 4; *p* = 0.059; *Z* = standardized residuals.*

The Pearson Chi-square statistical technique — used to compare the percentages of pupils given each sociometric status according to gender — failed to reveal a statistically significant value, although it is very close to significance. For this reason, the standardized residuals (corrected for the test) were interpreted. It is observed that in girls the average status type was recorded more frequently than in boys. In the other hand, the neglected status was more predominant in boys but not in girls.

### Psychosocial Adjustment and Its Relationship With Sociometric Status According to Gender

In this section we examine the association between the indicators of psychosocial adjustment of the SDQ and the different types of sociometric status for the entire sample (see Table 4). For these analyzes, those pupils with a controversial status were not taken into account, given their low number in the group. In general, data revealed that there are two opposing groups in terms of adjustment: popular versus rejected. In addition, the children in the rejected group show the poorest adjustment scores in comparison with those in the remaining sociometric status categories, with these differences being more marked for externalizing problems (conduct problems and hyperactivity). The children in the neglected status group presented a similar profile to those in the popular and average categories, except for emotional problems, where there were no differences from those in the rejected category.

**TABLE 4:** Sociometric status. Means (Standard Deviations) and F test of means based on SDQ measurements for the sample.

	**Popular**	**Rejected**	**Neglected**	**Average**	***F***	***η*^2^**
			
	***M* (*SD*)**	***M* (*SD*)**	***M (SD)***	***M* (*SD*)**		
Emotional problems	0.94 (1.13)^a^	2.58 (1.95)^b^	1.48 (1.95)^a,b^	1.57 (1.88)^a^	4.96**	0.060
Conduct problems	0.41 (0.91)^a^	3.00 (2.60)^b^	0.90 (1.68)^a^	1.28 (1.60)^a^	15.07***	0.162
Hyperactivity	1.47 (2.14)^a^	6.47 (3.10)^b^	2.60 (2.89)^a^	3.02 (2.85)^a^	21.05***	0.213
Peer problems	0.84 (1.17)^a^	2.61 (2.61)^b^	1.02 (1.59)^a^	1.25 (1.52)^a^	8.17***	0.095
Prosociality	9.16 (1.17)^a^	7.61 (2.14)^b^	8.64 (2.07)^a,b^	8.55 (1.94)^a,b^	3.96**	0.048

**p < 0.01; ***p < 0.001; η^2^ = eta-squared effect size. The different superscripts (a, b) indicate statistically significant differences. Test: Factorial ANOVA. Post hoc comparisons: Scheffé. No superscripts indicate that there are no statistically significant differences between groups.

These analyzes were replicated according to the gender of the pupils. The comparisons for boys and girls, respectively, are presented in Tables 5, 6. When the sample was separated by gender, the differences between the types of status in emotional symptomatology and prosocial behavior disappeared. For both genders, differences were found between the four types of status for hyperactivity, conduct problems, and peer problems, with boys and girls in the rejected groups presenting more adjustment problems than the rest of the groups, and with rejected boys exceeding the normal values established by the scale for hyperactivity and conduct problems (Goodman, 2001). As well, the differences between types of status in boys were of greater magnitude than in girls and were defined mainly by hyperactivity, whilst for girls the largest differences were evident in terms of conduct problems followed by hyperactivity.

**TABLE 5:** Sociometric status. Means (Standard Deviations) and F test for the comparison of means according to the SDQ measures for the sample of boys.

	**Popular**	**Rejected**	**Neglected**	**Average**	***F***	***η^2^***
			
	***M* (*SD*)**	***M* (*SD*)**	***M (SD)***	***M* (*SD*)**		
Emotional problems	1.06 (1.24)	2.70 (1.95)	1.56 (1.85)	1.81 (1.99)	2.53	0.067
Conduct problems	0.69 (1.20)^a^	3.45 (2.74)^b^	1.12 (1.81)^a^	1.60 (1.85)^a^	7.49***	0.176
Hyperactivity	1.44 (1.86)^a^	7.15 (3.08)^b^	2.64 (2.87)^a^	3.60 (2.97)^a^	14.46***	0.292
Peer problems	0.63 (0.96)^a^	2.55 (2.82)^b^	0.92 (1.15)^a^	1.50 (1.34)^a,b^	5.26**	0.131
Prosociality	9.13 (1.36)	7.20 (2.48)	8.24 (2.42)	8.21 (2.02)	2.46	0.066

**p < 0.01; ***p < 0.001; η^2^ = eta-squared effect size. The different superscripts (a, b) indicate statistically significant differences. Test: Factorial ANOVA. Post hoc comparisons: Scheffé. No superscripts indicate that there are no statistically significant differences between groups.

**TABLE 6:** Sociometric status. Means (Standard Deviations) and F test for the comparison of means according to the SDQ measures for the sample of girls.

	**Popular**	**Rejected**	**Neglected**	**Average**	***F***	**η^2^**
			
	***M* (*SD*)**	***M* (*SD*)**	***M (SD)***	***M* (*SD*)**		
Emotional problems	0.81 (1.05)	2.44 (2.00)	1.35 (2.15)	1.42 (1.81)	2.28	0.052
Conduct problems	0.13 (0.34)^a^	2.44 (2.37)^b^	0.59 (1.46)^a^	1.08 (1.39)^a^	7.34***	0.151
Hyperactivity	1.50 (2.45)^a^	5.63 (3.01)^b^	2.53 (3.01)^a^	2.67 (2.74)^a^	6.79***	0.141
Peer problems	1.06 (1.34)^a^	2.69 (2.41)^b^	1.18 (2.10)^a, b^	1.10 (1.61)^a^	3.74*	0.083
Prosociality	9.19 (0.98)	8.13 (1.54)	9.24 (1.25)	8.76 (1.88)	1.54	0.036

*p < 0.05; ***p < 0.001; η^2^ = eta-squared effect size. The different superscripts (a, b) indicate statistically significant differences. Test: Factorial ANOVA. Post hoc comparisons: Scheffé. No superscripts indicate that there are no statistically significant differences between groups.

## Discussion

Firstly, our study provides evidence of gender differences with respect to the psychosocial adjustment of pupils. Thus, girls stand out for having higher scores on prosocial behaviors, a finding that is compatible with those of previous studies (Plazas et al., 2010). In addition, boys have a greater number of conduct problems, which is in line with other international studies (Karreman et al., 2009; Chen, 2010), and those within our country (Aláez et al., 2000; Navarro-Pardo et al., 2012). However, our data differ from those of the latter studies in that we observe that externalizing problems are greater than internalizing problems in both boys and girls, as opposed to the finding that internalizing problems are more prevalent in girls. This finding could be explained by virtue of the fact that comparative studies often cover a wider age range — including adolescents — and internalizing problems reach higher levels (and are of greater frequency) in late childhood and during adolescence, particularly in girls (Jaureguizar et al., 2012). On the other hand, this result may be explained by the fact that children, parents, and teachers usually only report externalizing problems as adjustment problems, as these are regarded as annoying behaviors for others as opposed to internalizing problems that are not considered as adjustment problems in children of these ages (Del Rocío and Palos, 2005).

The distribution of the students across the sociometric status groups was as expected, with the majority being positioned in the average status group and the minority in the controversial status group (García-Bacete, 2008). With regard to gender, although this was a non-significant difference, we observed that boys were represented more in the neglected group compared with the girls who were represented more in the average group. Unlike previous studies (Plazas et al., 2010), we did not find that more boys were represented in the rejected group compared with girls. Finally, it should be noted that the low number of students in the controversial status group, as well as the problem of instability over time detected in previous studies (Newcomb et al., 1993), led us to exclude the data from these boys and girls in subsequent analyzes.

The association between sociometric status and psychosocial adjustment was also confirmed in this research. Firstly, the rejected status group presented an adjustment profile opposite to those in the popular status group. Furthermore, and in line with Mrug et al. (2012), rejection by peers was more strongly correlated with adjustment indicators than acceptance. Thus, we found that those in the rejected group differed from those in the rest of the sociometric status groups in almost all of the problems categories, with no such differences being found between the members of the remaining status groups. These differences were particularly marked for externalizing difficulties, especially hyperactive symptomatology. In this regard, a number of studies, as revealed by the meta-analysis carried out by Ros and Graziano (2018), show that children with ADHD experience a higher rate of rejection by their peers, which may be related to problems in the processing of social information, as well as difficulties in the self-regulation of both emotions and behavior.

We must also mention that the children in the neglected status group presented a similar profile to those in the popular and average categories, except for emotional symptomatology, where they do not differ from those in the rejected category.

The data obtained in this work also point to the possibility that the relationships between psychosocial adjustment and sociometric status should be analyzed differently for boys and girls, since the pattern of results found here differ according to whether we consider the sample as a whole or conduct separate analyses according to gender. This conclusion is consistent with other studies that have examined the moderating role of gender in the relationship between sociometric status and socio-emotional adjustment (Brendgen et al., 2005; Escobar et al., 2010).

Despite its strengths, the current work is not without limitations. Firstly, this is a cross-sectional study in which the fieldwork was conducted at a specific point in time. And whilst this has allowed us to analyze the variables considered in this work and the possible relationships between them, this type of design does not allow for establishing causal relationships that confirm the directionality of the relationships found, i.e., whether the adjustment problems determine the status of the children, or vice versa. Secondly, with regard to the participants, we did not use a clinical sample of children diagnosed with externalizing or internalizing problems, so the results might differ if a clinical and non-population sample was used. As well, it would be convenient to increase the size and the representativeness of the sample to verify whether the same results could be found in this case. Thirdly, we used self-report tests, in which the students themselves provided the information, whilst the teachers also completed a questionnaire. As demonstrated in the results of other studies, it would have been interesting to complement this evaluation with other questionnaires completed by the family. In this regard, and insofar as this is possible, it would be extremely useful to employ the same instruments for the pupils, parents/guardians, and teachers, in order to compare the different perceptions held by all parties on, for example, adjustment problems.

Finally, and to address these limitations, it would be interesting if future research could employ longitudinal designs that collect data throughout several educational stages, thereby allowing for an analysis of changes in the variables over time and to establish causal relationships between them. Also, some previous investigations have shown the heterogeneity that exists in some types of status (García-Bacete, 2008; Karmen and Tefan, 2013). So, it would be useful to identify different profiles or subtypes for each status, to know more in depth which profiles of each status are best related with indicators of personal and scholarly wellbeing.

In summary, as a final conclusion, the study provide evidence to suggest that rejection or acceptance by the peer group has important implications for the psychosocial adjustment of school age children, highlighting the need to develop interventions that could improve the social climate in the classroom (García-Bacete, 2008; Carrasco et al., 2015; Justicia-Arráez et al., 2015) and always being clear about the decisive role of teachers in this matter (Longobardi et al., 2019, 2020). Such interventions might be more effective at younger ages, given that, in comparison with older children, sociometric nominations are significantly less stable in younger children (Jiang and Cillessen, 2005).

### Practical Implications

Despite the prudence with which the results of a cross-sectional study are to be taken, our data point to the need to work with the classroom group in primary education to improve the wellbeing of children. So, the study highlights the support needs of boys and girls with a prominent profile in behavioral problems — and particularly those with hyperactive symptoms — to help with their integration and ability to adapt socially to their classmates. Taking into account the fact that this group is highly vulnerable to peer rejection, along with all the consequences that this entails for their development, it is essential to implement intervention programs focused on improving the sociometric status of these pupils (Mikami et al., 2013a). The program developed by these authors also had a significant impact on the peers of children with ADHD, since this intervention also decreased their social problems, improved their sociometric status, and allowed them to achieve greater reciprocity in their friendships. Moreover, these effects were more significant for those children with higher levels of disruptive behavior (Mikami et al., 2013b). Taken together, all of this confirms the usefulness of these types of programs for improving coexistence in the classrooms and the subsequent psychological wellbeing of all the pupils, but particularly of those with psychosocial adjustment problems. In this sense, we must underline the important role of teachers, since various studies have highlighted the protective role of the teacher-student relationship with respect to psychological symptoms, in addition to promoting prosocial behaviors, academic outcomes, school adjustment and general psychological well-being of their students (Longobardi et al., 2019, 2020).

For all the above mentioned, and in agreement with other authors (Salerno, 2016; Mundy et al., 2017) we focus the need of connection between education policy and awareness and promotion of mental health in primary school children. Basically, through programs that emphasize social and emotional skills to the prevention of both academic failure and school disengagement as more serious mental health problems in adolescence and adulthood.

## Data Availability Statement

The raw data supporting the conclusions of this article will be made available by the authors, without undue reservation, to any qualified researcher.

## Ethics Statement

Ethical approval was not provided for this study on human participants because no patients were included in this study and no ethics committee approval was needed. Written informed consent to participate in this study was provided by the participants’ legal guardian/next of kin.

## Author Contributions

All authors listed have made a substantial, direct and intellectual contribution to the work, and approved it for publication.

## Conflict of Interest

The authors declare that the research was conducted in the absence of any commercial or financial relationships that could be construed as a potential conflict of interest.

## Footnotes

1 http://www.sdqinfo.org/py/sdqinfo/f0.py
